# Analysis of seroprevalence in target wildlife during the oral rabies vaccination programme in Lithuania

**DOI:** 10.1186/s13028-021-00577-z

**Published:** 2021-03-20

**Authors:** Dainius Zienius, Janina Mickutė, Arnoldas Pautienius, Juozas Grigas, Arunas Stankevičius, Gediminas Pridotkas, Eugenijus Jacevičius, Jolita Kemeraitė, Ingrida Jacevičienė

**Affiliations:** 1 Lithuanian University of Health Sciences, Institute of Microbiology and Virology, Lithuania, Tilžės str. 18, 47181 Kaunas, Lithuania; 2 National Food and Veterinary Risk Assessment Institute, National Food and Veterinary Risk Assessment Institute, J. Kairiūkščio str. 10, 08409 Vilnius, Lithuania

**Keywords:** Rabies, Lithuania, Raccoon dog, Red fox, ELISA, Seroconversion, Vaccination

## Abstract

**Background:**

Rabies vaccination of wildlife carnivores is a powerful tool to prevent, control and eliminate rabies. The presence of neutralizing rabies antibodies in blood is considered a reliable indicator of adequate vaccination. The main purpose of the present study was to analyze the seroprevalence of specific antibodies in target populations of Lithuanian red fox (RF) and raccoon dog (RD) during the oral rabies vaccination (ORV) campaigns during the 2010–2019 period.

**Results:**

Over the ten-year period, 7,261 RF and 2,146 RD sera samples were collected post-mortem in field conditions and tested using a commercial standardized enzyme-linked immunosorbent assay (ELISA) kit in Lithuania. In the ORV spring and autumn vaccination periods, 31.8% (20.3–43.4 95% CI – 95% confidence interval) and 31.7% (21.2–42.1 95% CI) of RF, and 34.1% (22.5–45.7 95% CI) and 34.7% (22.7–46.7 95% CI) of RD sera samples, respectively, were identified as ELISA-positive (seroconversion ≥ 0.5 EU/mL—Equivalent Units per Millilitre). The seroprevalence analysis in adult/ juvenile animal subpopulations indicated that 34.9% (27.2–42.5 95% CI) and 29.2% (20.3–37.9 95% CI) of RF, and 35.6% (25.2–46.0 95% CI) and 30.6% (20.2–40.9 95% CI) of RD sera samples, respectively, were identified as ELISA-positive (seroconversion ≥ 0.5 EU/mL). Statistically strong determinate correlations (*r*) between the serological results (pos.%) in RF adult/juvenile animal subpopulations (*r* = 0.937) and between RF and RD positive seroconvert (pos.%) sera samples during the spring vaccinations (*r* = 0.864) were demonstrated. In different ORV periods, 14–29% of RF and 7–25% of RD sera samples were identified as ELISA-negative (seroconversion < 0.5 EU/mL), but with low (0.125 < 0.49 EU/mL) antibody (Abs) titres.

**Conclusions:**

The 2010–2019 ORV programme has been an effective tool in both RF and RD populations in Lithuania. The rabies-free status of Lithuania was self-declared in 2015 with only three rabies cases identified in buffer zones since then. The percentage of ELISA-positive serum samples (seroconversion ≥ 0.5 EU/mL) during the different periods of vaccination was similar in RF and RD populations—32% and 34% respectively. The identified seroconversion average of 21.5% in RF and 16% in RD sera samples were officially identified as ELISA-negative (seronversion < 0.5 EU/mL), but with low 0.125 < 0.49 EU/mL Abs titres. That low, but positive seroconversion participated in the formation of populations overall immune status and can influence the interpretation of oral vaccination efficacy.

## Background

Laboratory and epidemiological investigations of rabies cases in wildlife show that red foxes (RF, *Vulpes vulpes*) and raccoon dogs (RD, *Nyctereutes procyonoides*) are relatively susceptible to the classical strains of rabies virus (RV) in Europe and constitute a possible inter-species transfer pathway in the wild [1]. A long-term retrospective study of the rabies epidemiological status in wildlife in eastern and northern regions of Europe identified that rabies-positive cases have been observed mainly in RF and RD [2, 3]**.** The involvement of two alternative rabies vectors in the epidemiological chain has challenged health authorities and cast doubts on the success of the traditional method of oral rabies vaccination (ORV), which has been used successfully to control rabies (targeting RF) in western Europe [4]. However, a retrospective analysis of ORV programmes has shown that despite the adaptive capacity of rabies viruses and the potential for inter-species transmission, this natural infection can be controlled in wildlife using the classic control tool of oral vaccination [5]. Therefore, with financial support from the European Union (EU), ORV programmes are currently being implemented in regions of 12 EU member states (including the Baltic countries), as well as in neighboring countries such as Russia, Ukraine, Belarus and the Western Balkans [4, 6].

Almost all the ORV campaigns that have been carried out are being monitored and their effectiveness in field conditions evaluated. These studies are being conducted under national programmes for the control of effectiveness of rabies vaccination and are based on direct and indirect assessment methods, including quantitative testing of oral vaccine bait distribution in target populations (bait uptake), evaluation of post-vaccination immunological status (seroprevalence in target species), and epidemiological studies of rabies positive/ negative cases in wildlife (post-vaccination period) [6, 7].

Detection of rabies virus neutralizing antibodies (Abs) is one of the key factors in determining successful vaccination [8, 9], leading to a positive evaluation of rabies vaccination efficacy [10, 11]. The World Health Organization [12] and the World Organization for Animal Health [13] recommend two reference methods that allow quantification of specific neutralizing Abs against RV [14, 15]: the fluorescent antibody virus neutralization test (FAVN) [16] and the rapid fluorescent focus inhibition test (RFFIT) [17]. Both methods have been used for a considerable time in rabies reference laboratories as a gold standard in the measurement of Abs. They are of sufficient quality and reliability because they are based on the classic seroneutralization of live RV in sensitive cell systems. Nevertheless, both methods are relatively expensive, take a long time to complete, and require highly specialized laboratory technicians and high biosecurity-level implementation. Moreover, methods are based on susceptible cell cultures and the success of testing is highly dependent on the quality of the test samples, which may contain cytotoxic substances and contaminants that directly influence the sensitivity, quality and prognosis of the methods [18, 19]. This is especially true when it comes to measuring the effectiveness of ORVs in wildlife and using serological testing of field samples, which are classified as “body fluids” rather than clean sera samples.

Therefore, to assess the status of rabies post-vaccine immunity in the field, a new serological assay was required that was better suited to poor-quality, haemolysed, relatively cytotoxic and potentially contaminated wildlife specimens [20]. Thus, a method significantly less sensitive to external factors for rabies serological testing—the enzyme-linked immunosorbent assay (ELISA)—has been proposed. ELISA is quick and easy to perform, allowing for large-scale testing which is particularly important for controlling the effectiveness of ORV programmes. This method is not subject to strict biosafety requirements because live RV and sensitive cell cultures are not used to perform the test [21]. ELISA has repeatedly been used to detect rabies-specific Abs in domestic/ wild animal sera samples of different biological quality, and can be considered as one of the most acceptable methods for monitoring the efficacy of ORV campaigns in foxes for avoiding cytotoxicity problems [6, 18, 22, 23]. Bio-Rad (Marnes-La-Coquette, France) developed an ELISA kit (Platelia™ Rabies II kit *ad usum veterinarium*) for serum samples of domestic and wild carnivores over twelve years ago. The method was approved by ANSES Nancy Laboratory [24] and evaluated before marketing [22]. Eight EU countries running national ORV programmes have adopted an ELISA to evaluate vaccine effectiveness in wildlife [6]. These investigations reveal high variability in serological results, with a specific antibody response ranging from 17 to 82% [6]. Varying investigation results have initiated a broad scientific debate on evaluation of the efficacy of post-vaccine immunity status and the priorities for the control of oral vaccination programmes in wildlife.

The objective of the present study was to analyze the status of seroconversion of Lithuanian ORV campaigns during the 2010–2019 period using seroprevalence studies of rabies-specific Abs in RD and RF sera samples collected from vaccination areas in the field.

## Methods

### ORV

Oral vaccination of wild animals against rabies using baits with attenuated vaccine started in 2006 and has been systematically carried out in Lithuania according to the classic scheme of bait distribution [25–27]. In the 2006–2015 period, spring (March–May) and autumn (October–December) ORV campaigns were carried out in wildlife throughout the country (65,000 km^2^). Vaccine baits were dropped from aircraft manually and using mechanical devices, with an average concentration of 20 baits/km^2^ in all RF and RD inhabited areas. Given that the 2006–2015 ORV programme in Lithuania was effective and the epidemiological situation of rabies improved, since the spring of 2016 vaccinations have been carried out according to the new programme approved by the European Commission [5]. Under this programme, vaccine baits have not been used throughout Lithuania, but are only distributed at its borders (buffer zones) with neighboring countries: the Kaliningrad region of the Russian Federation, Poland and Belarus. Thus in line with the newly implemented Programme for Rabies eradication submitted for obtaining EU co-financing, in the 2016–2019 period, spring (March–May) and autumn (October–December) ORV campaigns were carried out in wildlife in the buffer zones with neighboring countries (Fig. 3). 1,715,000 vaccine baits were distributed every year in the 35,300 km^2^ vaccination area. To increase the effectiveness of the programme, vaccine baits in these areas have been thrown at twice the density – with an average concentration of 48 baits/km^2^ in all the selected RF and RD inhabited areas.

### Vaccine

LYSVULPEN (Bioveta A.S., Czech Republic) live attenuated RV vaccine (SAD-Bern strain, biological activity 1.8 × 10^6^ TCID_50_/bait; TCID – tissue culture infective dose) was used in ORV programmes in Lithuania in the periods 2006–2010 and 2013–2019. During the 2011–2012 ORV program FUCHSORAL (IDT Biologika GmbH, Germany) vaccine (live SAD B19 strain with biological activity 10^6^ FFU/mL; FFU—focus forming units) was used in Lithuania. Vaccines were stored according to the instruction manuals in refrigerators at/below − 20 °C before use and throughout the vaccination campaigns.

### Sample collection

To evaluate the effectiveness of ORV campaigns, recommended sampling schemes (EFSA/WHO) in vaccination regions were used, taking into account the investigation of post-vaccination seropositive status in target populations [7, 12, 28, 29]. For rabies-specific antibody seroprevalence testing, samples of target animals (RF and RD) were collected throughout the ten-year period, covering the country’s entire territory and at all stages of oral vaccination, regardless of the vaccination period. The RF and RD serum samples were collected under field condition throughout the all-year hunting periods and evaluated in two time-interval categories. Samples collected during January-June and during July-December were tested and evaluated as spring (S) ORV and as autumn (A) ORV samples, respectively. Of the samples tested, 80% were from hunted animals, about 15% were from animals found dead in the wild, and the remaining 5% were from animals that had died of other causes. Target blood samples (blood clots, thoracic transudates) were collected under field conditions directly from the chest cavity and placed in a refrigerator at + 4 °C for 24 h. Sera samples were inactivated (30 min at 56 ± 2 °C) and stored at − 20 °C until testing. All the collected samples (independent of their quality) were analyzed for specific rabies Abs by ELISA at the National Food and Veterinary Risk Assessment Institute of Lithuania (NFVRAI).

### ELISA

Commercial standardized ELISA kits (Platelia™ Rabies II kit *ad Usum Veterinarium*, Bio-Rad, France) were used for RV-specific Abs detection and titre evaluation of blood sera samples from animals. An assay was performed in a standard 96-well microplate coated with RV glycoprotein, as previously described [24] and according to the manufacturer's instructions. Briefly, diluted (1/100) samples (100 µL), calibrated positive controls (PC) or quantitative standards (S1–S6) in duplicates were distributed in microplate wells and incubated for 1 h at 37 ± 2 °C. After washing (3 ×), conjugate (100 µL Protein A-peroxydase) was added and incubated for 1 h at 37 ± 2 °C. Following incubation, microplate washings were performed again (5 ×), the substrate (100 µL of TMB chromogen solution) was added, and the microplate was incubated in the dark at room temperature for 30 ± 5 min. After that, blocking solution (100 µL H_2_SO_4_ sol.) was added to the microplate wells to stop the reaction. Optical density (OD) was measured using an automatic microplate analyzer (spectrophotometer) Elx808 (Bio-Tek, USA) at 450 nm wavelength. The positivity threshold was determined using control sera provided with the diagnostic kit. The standard curve was established using the OD values obtained for each quantification standard (S1-S6) according to the manufacturer’s instructions. Antibody titres in quantitative assays were obtained after direct reading of the standard curve and expressed in equivalent units per millilitre (EU/mL), which was calculated using the standard linear regression method. Results were evaluated when the negative control OD value was less than 0.05, the R4a positive control OD value was between 0.300 and 1.200, and the R4b positive control OD value was between 1.500 and 3.500. The threshold was the mean of two R4a positive controls (OD R4a) and corresponded to a protective value of 0.5 EU/mL [4, 9, 28, 30]. The high protection value equaled the average of two R4b positive controls (OD R4b). The optical density of each sample was compared with the high protection and threshold values. The seroconversion rate of the ELISA test samples was identified in four categories depending on the determined Abs titres: negative (< 0.125 EU/mL); low (0.125 < 0.49 EU/mL), standard (0.5 ≤ 2 EU/mL) and high (> 2 EU/mL). The laboratory test results from all these different seroconversion groups were used for statistical analysis.

## Statistical analysis

Statistical data analysis was performed using IBM SPSS Statistics Base (IBM, USA). Statistical calculations of positive (Abs titers of 0.5 ≤ 2 EU/mL and > 2 EU/mL) and negative (Abs titers < 0.125 EU/mL and 0.125 < 0.49 EU/mL) ELISA samples (n/%) were reported. General statistical accounting methods were used and mean (M), minimum and maximum values (Min/Max), average standard deviation (± SD) and 95% confidence interval (95% CI) were counted for data analysis and interpretation. One-Way ANOVA procedure and Post Hoc Multiple Comparisons types of tests for comparing M was used. Tukey’s significant difference range statistic tests for multiple pair wise comparisons between groups were chosen. The comparative statistical analyses were performed in each study category: RF and RD populations, juvenile and adult subpopulations, spring and autumn vaccination periods. To evaluate the association between different categories rank orders the Bivariate Correlations procedure was used, Spearman correlation coefficient (*r*) was calculated and analyzed using a confidence factor (*p*).

## Results

**Fig. 1:**
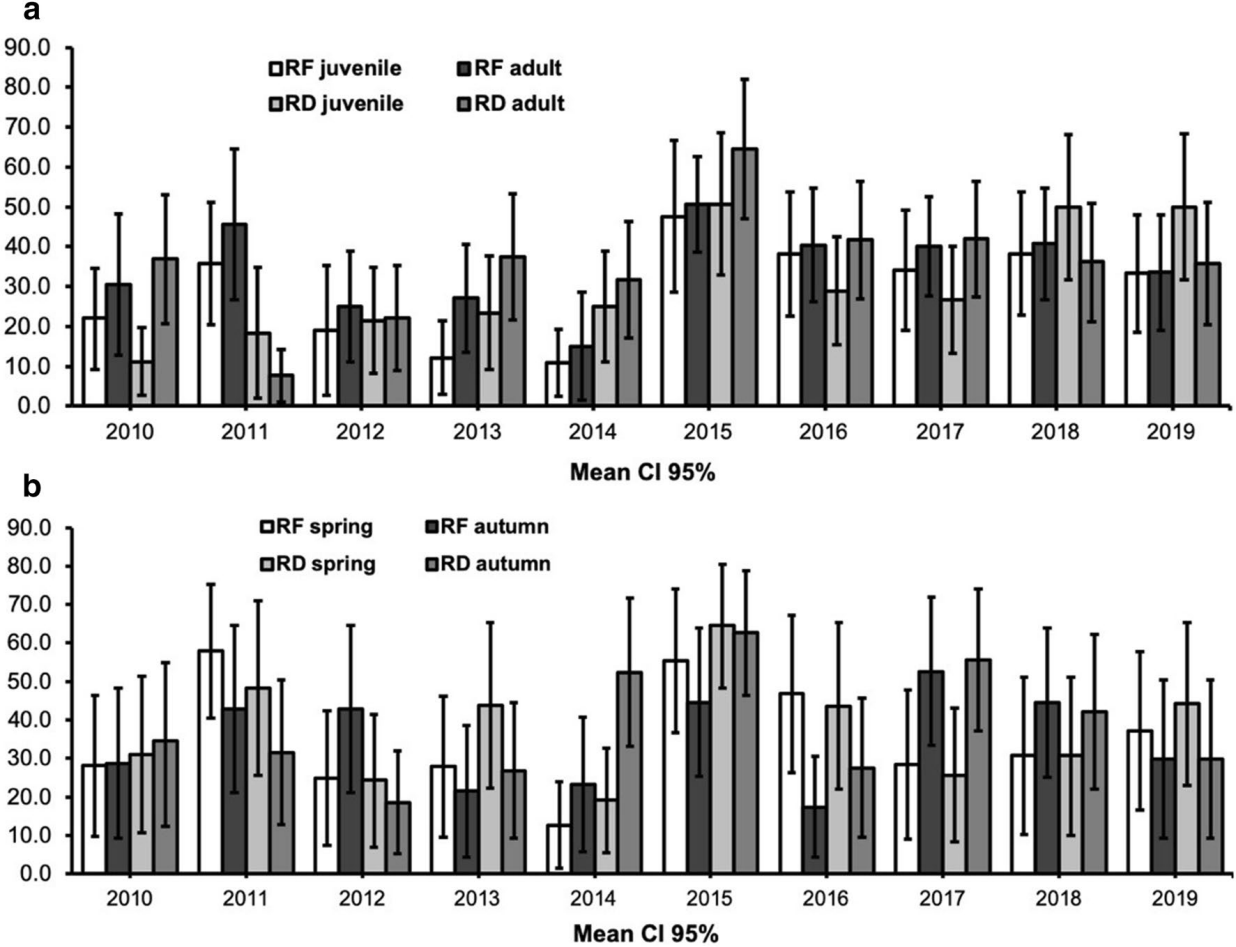
Comparative analysis of 2010–2019 ORV seroconversion (pos.% at ≥ 0.5 EU/mL) in different age groups of red foxes (RF) and raccoon dogs (RD) (**a**), and seroconversion (pos.% at ≥ 0.5 EU/mL) of red foxes and raccoon dogs in the 2010–2019 ORV spring and autumn campaigns (**b**)

During the 2010–2019 ORV programme, 2,214 of 6,345 (34.9%) tested adult RF (27.2–42.5 95% CI); Additional file 1) and 641 of 1,800 (35.6%) tested adult RD (25.2–46.0 95% CI Additional file 2) sera samples were ELISA-positive (seroconversion ≥ 0.5 EU/mL; Fig. 1a). The same investigation in a category of young animals showed 266 of 916 (29.2%) tested juvenile RF (20.3–37.9 95% CI; Additional file 1) and 106 of 346 (30.6%) tested juvenile RD (20.2–40.9 95% CI; Additional file 2) were identified as ELISA-positive (Fig. 1a). Annual immunization rates (pos.%; Fig. 1a) in the adult animal subpopulation ranged from 15.0% (1.6–28.5 95% CI; 2014) to 50.6% (38.6–62.6 95% CI; 2015) in RF and from 22.0% (8.9–35.2 95% CI; 2012) to 64.6% (47.1–82.1 95% CI; 2015) in RD (Additional file 3). The same statistical calculation rates in the young animal subpopulation ranged from 10.8% (2.4–19.2 95% CI; 2014) to 47.6% (28.6–66.7 95% CI; 2015) in juvenile RF and from 11.2% (2.6–19.8 95% CI; 2010) to 50.7% (32.9–68.7 95% CI; 2015) in juvenile RD (Additional file 3). The comparative statistical analysis between adults and juvenile animal subpopulations (pos.%) identified a strong positive correlation (*r* = 0.937, *p* = 0,0001) in the RF population, with an average standard deviation SD of ± 5.75%. The same statistical comparison in the RD population showed a moderate correlation (*r* = 0.538, *p* = 0.054) between adults and juvenile animals (pos.%), with an SD of ± 4.04%.

The investigation of specific Abs in RF and RD blood sera samples using ELISA during the 2010–2019 ORV spring vaccination periods (Fig. 1b) showed that 1190 RF of 3741 (31.8%) tested (20.3–43.4 95% CI; Additional file 4) and 292 RD of 857 (34.1%) tested (22.5–45.7 95% CI; Additional file 5) sera samples were identified as ELISA-positive (seroconversion ≥ 0.5 EU/mL). Annual immunization rates (pos.%) during the spring vaccination periods (Fig. 1b) ranged from 12.8% (1.5–20.4 95% CI; 2014) to 58.0% (40.8–75.4 95% CI; 2011) in RF and from 19.1% (5.4–32.8 95% CI; 2014) to 64.5% (48.5–80.6 95% CI; 2015) in RD (Additional file 6). In the same ORV autumn periods (Fig. 1b), of the 3520 RF and 1,289 RD sera samples investigated, 1156 RF (31.7%, 21.2–42.1 95% CI Additional file 4) and 447 RD (34.7%, 22.7–46.7 95% CI Additional file 5) were identified as ELISA-positive (seroconversion ≥ 0.5 EU/mL). Annual immunization rates (pos.%) during the autumn vaccination periods ranged from 17.4% (4.3–30.5 95% CI; 2016) to 52.6% (33.3–71.9 95% CI; 2017) in RF (Additional file 6), and from 18.5% (5.1–31.8 95% CI; 2012) to 62.6% (46.3–78.9 95% CI; 2015) in RD populations (Additional file 6). The comparative statistical analysis between spring and autumn vaccination periods identified weak correlations in both (RF and RD) study groups. The correlation coefficient in the RF population was *r* = 0.353 (*p* = 0.159), and the same statistical comparison in the RD population showed an even smaller correlation coefficient (*r* = 0.102, *p* = 0.390) between ELISA-positive samples in the spring and autumn vaccination periods.

**Fig. 2:**
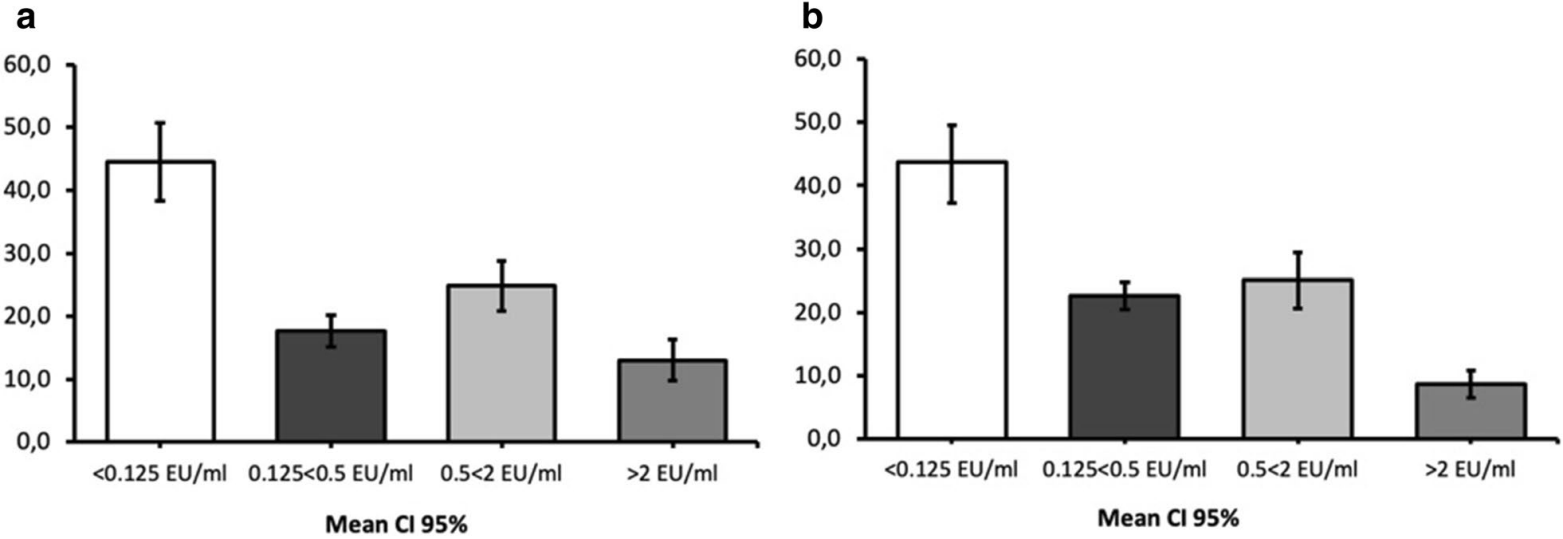
Seroconversion (ELISA Abs titres EU/mL,%) in Lithuanian raccoon dogs (**a**) and red foxes (**b**) during the 2010–2019 ORV vaccination period

The investigation of specific Abs in RD and RF blood sera samples using ELISA during the 2010–2019 ORV programme indicated that 44.5% (38.3–50.7 95% CI; Fig. 2a) RD and 43.4% (37.2–49.6 95% CI; Fig. 2b) RF serum samples were negative (< 0.125 EU/mL). However, in the same ORV period, 22.6% (20.4–24.8 95% CI; Additional file 4) RF and 17.6% (15.1–20.2 95% CI; Additional file 5) RD serum samples with low specific antibody titres (0.125 < 0.49 EU/mL) were detected, having also been officially classified as negative. ELISA antibody titres greater than 2 EU/mL were detected in 8.6% (6.4–10.8 95% CI; Additional file 4) RF and 13.0% (9.7–16.4 95% CI; Additional file 5) RD serum samples.

A comparative statistical calculation (RF vs. RD) in the period of ORV was performed in two categories of investigation: juvenile/adult subpopulations (Fig. 1a; Additional file 3) and spring/autumn vaccination periods (Fig. 1b; Additional file 6). In the 2010–2019 ORV spring vaccination periods, 3741 of 7261 RF sera samples (51.5%) and 857 of 2146 (39.9%) RD sera samples were ELISA tested. The comparison between RF and RD seropositive sera samples during the spring vaccination period revealed a strong determinate linear correlation (*r* = 0.864, *p* = 0.0006), with an SD of ± 9.53%. In contrast to the spring vaccination periods, a weak statistical relationship between RF and RD populations was observed in the autumn periods, with a weak positive linear correlation (*r* = 0.388, *p* = 0.134) and an SD of ± 2.34%.

During the ORV programme period, 87.2% (with 34.9% positive) of ELISA-tested RF and 83.7% (with 35.6% positive) of RD sera samples were collected from adult animals. The comparison between RF and RD positive samples in the adult animal subpopulation (Additional file 3) identified a weak linear correlation (*r* = 0.299, *p* = 0.200), with an SD of ± 1.27%. In contrast to adult animals, a reasonably strong linear correlation (*r* = 0.603, *p* = 0.032) was observed between RF and RD juvenile subpopulations, with an SD of ± 4.03%.

**Fig. 3:**
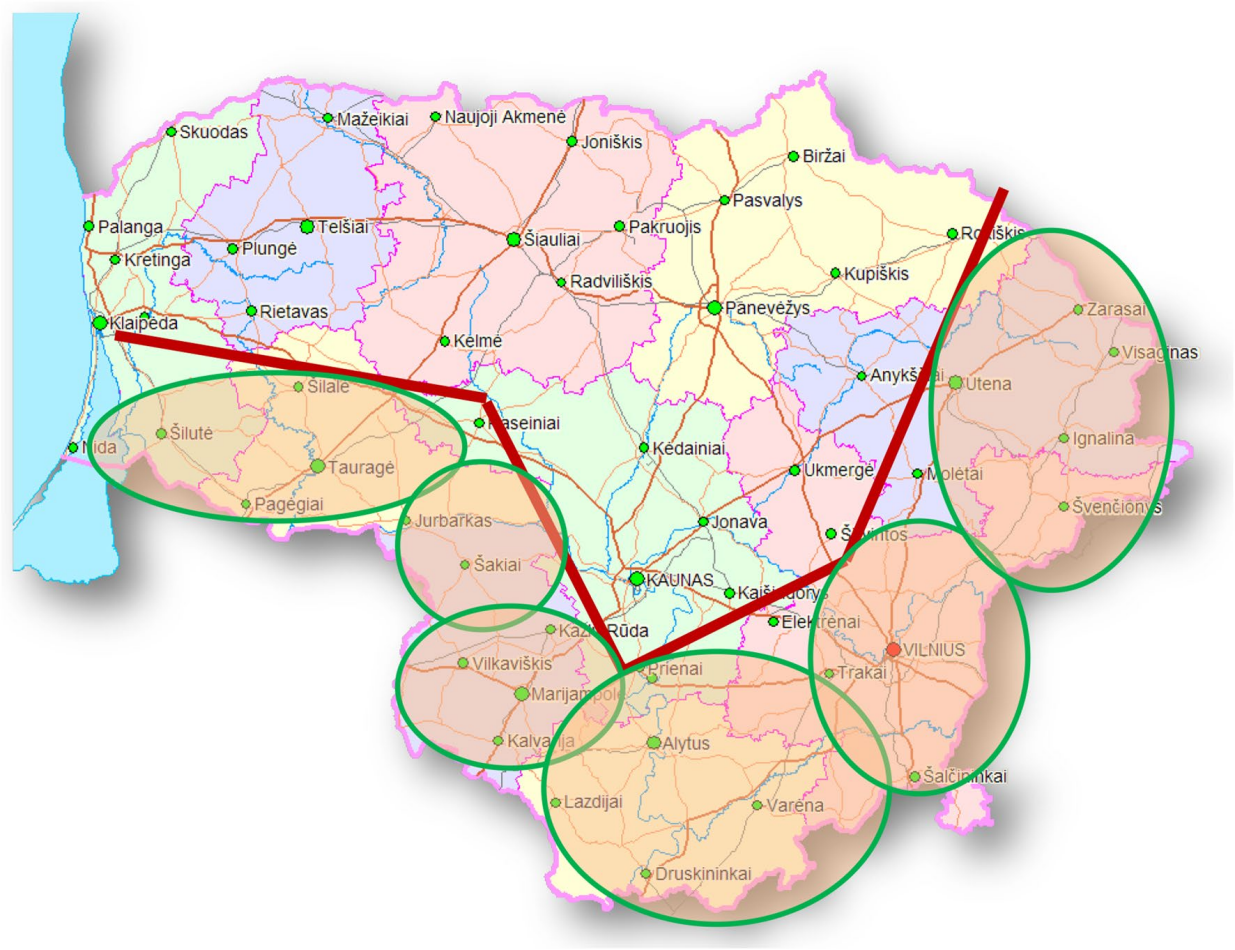
2016–2019 ORV program in Lithuanian buffer zone (35.300 km^2^ vaccination area). National programme for rabies eradication submitted for obtaining EU co-financing

During the 2016–2019 ORV period 3,346 of tested 7261 RF serum samples and 809 of tested 2,146 RD serum samples were collected in the vaccination protection zone (Fig. 3). The investigation of RF annual immunization rates (pos.%) during the different years of vaccination periods showed that the highest seroconversion was found in 2017 (44.5%) and the smallest in 2018 (29.8%). The same investigation of sera samples from vaccination protection zone in RD population identified the highest seroconversion in 2016 (38.5%) and the lowest in 2019 (21.8%). Comparative analysis of seroconversion rates between the samples collected during 2016–2019 period in the vaccination protection zone and the sample collected in the rest of the territory of Lithuania identified a statistically minute difference between the seroconversion averages in these regions. Annual immunization rates (pos.%) in RF sera samples from the vaccination protection zone was 37.5% vs seroconversion of 31.3% in sera samples from other territories of Lithuania. The same comparative analysis in RD population revealed annual immunization rates of 30.7% (protection zone) vs 28.6% (other territories). Comparative study of seroconversion rates (pos.%) between the vaccination protection zone and the rest of Lithuanian territory showed that the mean of seroconversion rates in RF sera samples from vaccination zone were significantly (*p* = 0.046) higher at 7.5%.

**Fig. 4:**
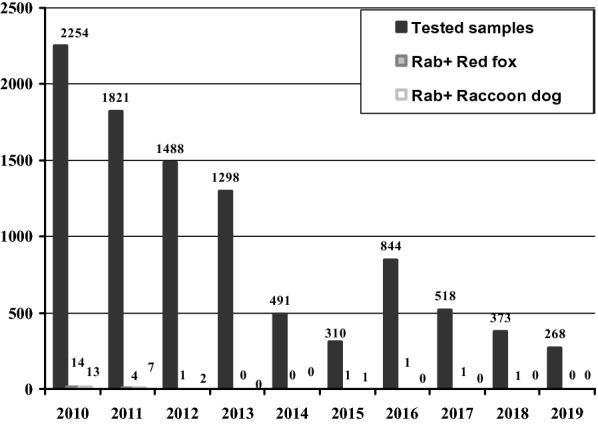
The positive Rabies cases per year in Lithuanian red fox and raccoon dog populations in 2010–2019

Epidemiological data (one of the main effectiveness criteria) during the implementation of the ORV program are very important for evaluating of the ORV program. In Lithuania, 14 rabies-positive cases (Rab. pos.) were detected in the RF population and 13 in the RD population in 2010, while in 2013 and 2014, no rabies-positive cases were detected (Fig. 4). Since 2015 (confirmed rabies-free status), one positive case of rabies per year (2016–2018) was detected in RF population in the buffer vaccination zone. Therefore, such rabies epidemiological data confirm the efficacy of Lithuanain ORV during the study period.

## Discussion

In the last three decades, the implementation of ORV programmes in 24 countries has led to eradication of fox-mediated rabies from large areas of western and central Europe [26, 31, 32]*.* ELISA serological testing of vaccinated wild animals has been widely performed throughout Europe, especially for evaluating ORV efficacy in vaccinated fox populations [33]. ELISA has been used to optimize common test protocols between reference laboratories in different countries (the first inter-laboratory testing was conducted in 2003) for comparative analysis of seroprevalence results [18]. In 2010, 73% of national rabies reference laboratories in the EU used commercially available ELISA kits to test ORV vaccination effectiveness, with 18% and 9% of reference laboratories using RFFIT and FAVN tests respectively [29, 34].

For a long time in many ORV programs, the target wildlife for vaccination has been RF, however, in the last 30–35 years in northern Europe (including Lithuania) RD has become an important co-factor in the transmission of rabies. These two species are particularly important in the comparative analysis and interpretation of ORV seroconversion rate in Lithuanian wildlife populations. In the 2010–2019 ORV period in Lithuania the comparative seroconversion analysis between RF and RD was performed, because these two species have “slightly” different ecological cycles in their identical area of habitation (subsequent reproductive cycle of RD). The data of seroconversion in spring/autumn ORV therefore was of particularly importance (strong correlation in the seropositive % between RF and RD during the spring vaccinations). The seroconversion in adult and juvenile age groups was analyzed because the formation of long-term population immune status (including multiple vaccinations) as a consequence of ORV can only be assessed relatively, as the age-related structural indicators of the target populations can vary greatly long term (strong correlation between the seropositive % in RF adult/juvenile animal subpopulations). The statistical investigation of ELISA-positive (seroconversion ≥ 0.5 EU/mL) sera samples in juvenile subpopulations (RD vs RF) showed that the variations in seroconversion rates (pos.%) during the analytic period were similar in both species (10.8–47.6% RF and 11.2–50.7% RD). However, during the last 3 years of the ORV program, seroconversion in the RD juvenile population was significantly higher compared to RF (42.3% RD vs 35.2% RF). Juveniles (cubs) of both species are not the targets of spring ORV (March–May). The priority of the spring ORV is immunization (“booster” vaccination) of young animal (12–16 months old) subpopulations that may have been vaccinated in the last year autumn (October-December) ORV campaigns.

During the ORV study period the average seroprevalence rate (positive% ≥ 0.5 EU/mL) was approximately 33% in RF and 38% in RD populations in all age groups. At the same time, large variations in seroconversion levels (seropositive %) were observed in Lithuania; of ELISA-positive samples, 28% and 33% were identified in 2010 for RF and RD, respectively (four years after the ORV commenced). However, in 2015 there were 50% positive samples in RF and 64% in RD sera. It should be noted that similar variations have been found in the seroprevalence rates of target red fox and raccoon dog sera samples in the other Baltic countries, with the largest seropositive sera samples observed in Latvia (73%—autumn 2010) and the smallest in Estonia (30%—autumn 2011) [4]. Possible reasons for these seroprevalence results can be associated with the poor quality of the sera samples collected under field conditions, ELISA sensitivity as well as the ecological factors of target wild animals.

Analysis of long-term national ORV programmes under field conditions showed that the overall bait consumption (TTC marker) was 80%, while the seroprevalence rate was approximately 50% [3, 26, 29]. A relatively large discrepancy between bait uptake and seroprevalence was attributed to a variation in the sensitivity of the commercially available ELISA kits due to the poor quality of field samples (haemolysis, bacterial contamination) compared with blood samples from experimentally vaccinated RF and RD [26, 35]. Many studies have identified the influence of poor-quality serum samples on ELISA quality. One possible reason might be associated with partial denaturation (degradation) of IgG heavy chains in poor-quality tissue fluids and heavily haemolyzed blood samples collected post-mortem in the field [6, 35, 36]. During the investigation of Lithuania’s ORV programme, 25–40% (depending on the year, hunting period, sample collection and transportation conditions, species etc.) of samples were highly haemolyzed. Some of them (10–25%) could be categorized as “body or tissue fluids” and theoretically would not qualify for testing by the spectrophotometrical optical density ELISA method. However, all the collected haemolyzed sera samples were investigated and approximately 20% of them were identified as positive (with RV Abs titers of 0.5 ≤ 2 EU/mL), and 12–15% were identified as negative, but with low titers (0.125 < 0.49 EU/mL). The high seroconversion (with RV Abs titres of > 2 EU/mL) were not detected in hemolyzed serum samples. A certain degree of variability in the quality of sera should be expected under field conditions. Any test must therefore be robust enough to avoid the false positive/negative results due to serum quality [35]**.**

Similar differences in ELISA sensitivity have been determined by comparing Bio-Rad ELISA Platelia™ Rabies II kit (which was used during the 2010–2019 ORV program in Lithuania) and another commercially available ELISA kit (BioPro ELISA kit) in animal blood sera samples. The results showed greater sensitivity in the BioPro ELISA kit (83.2%) compared with the Bio-Rad Platelia™ Rabies II kit (66.4%) [37]. The observed differences may be related to the lower sensitivity of the Platelia Rabies II kit, previously reported in field-collected fox sera [35], and can be associated with difficulties in interpreting oral vaccination data [21]. However, in Latvia, where both ELISA kits (Bio-Rad and BioPro) have been used to test seroprevalence during the ORV programme in wildlife, the investigation of kit sensitivity identified a high degree of differences of Abs titres. BioPro ELISA results showed lower seroconversion rates than the Bio-Rad ELISA kit, therefore the results of that study were completely different (converse) to the data provided by other studies [4, 21, 37]. These results may have been influenced by the use of a lower positive cut-off value concentration (0.125 EU/mL) than the recommended (0.5 EU/mL) cut-off value concentration of Abs titres in the Bio-Rad kit, which was probably too restrictive. In fact, using a threshold level of 0.2 and 0.3 EU/mL led to the observation of a significant increase in ELISA sensitivity (92.68% and 86.87% respectively) and better correlation with FAVN [9]. Therefore, the different commercial ELISA kits (Bio-Rad/ BioPro) potentially initiate different seropositive results not only due to the biochemical properties of the rabies glycoprotein (purity/degradation), but also due to different optical density evaluation criteria. The use of different ELISA kits may influence the objective result (seropositive %), their interpretation and as a consequence—ORV efficacy in analytic/prognostic models.

There are various different opinions about the minimum reliable RV-specific Abs titres in vaccinated wildlife that would suggest a minimal protective immune response in a susceptible population. Understandably, the Abs level is one of the main indicators of resistance to infection, however the statistical evaluation of seroconversion in different biological models is not absolutely correct, i.e., some animals with “sufficient” or even “high” Abs titres may potentially become infected with RV and some seronegative individuals might survive after experimental virus infection [38–41]. During the time of investigation of the Lithuanian ORV, more than 1,100 RF and 400 RD sera samples were identified with low 0.125 < 0.49 EU/mL Abs titres (positive, but insufficient serological response), and were qualified as Bio-Rad ELISA-negative (< 0.5 EU/mL). In the spring ORV periods, 23% RF and 18% RD sera samples with low Abs titres were identified. Similar results were found during the autumn ORV periods, with 21% RF and 17% RD sera samples with 01.25 < 0.49 EU/mL Abs titres. The same proportion with 1–2% variation of ELISA-negative samples was acquired in juvenile/ adult target animal subpopulations. About 10% of ELISA seronegative samples had antibody titers of 0.4–0.49 EU/mL, therefore positive seroconversion participated directly in the formation of overall immune status of the population. According to the age statistic of hunted animals, 8–10% of older (> 5 years) animals are hunted in Lithuania. During the different Lithuanian ORV periods, 70–80% of in field-collected samples subjected to ELISA were from animals 1 to 3 years of age. Therefore the formation of long-term immune status of the population (including the multiple vaccination) as a consequence of ORV can only be assessed relatively, as the age-related structural indicators of the target populations can vary greatly on a long-term basis. Under the influence of different ecological/epidemiological/reproductive factors, the age-related structural change of the RF/RD populations can reach 50% and more in different time periods. Thus, the 35% statistical average of ORV seroprevalence status in susceptible animal populations may already be a “constant-attractive” factor in epidemiological control. Most discrepancies were observed in serum samples with antibody titres close to the ELISA threshold value (0.5 EU/mL). Serum samples with antibody titres between 0.5 and 1 EU/mL were found to have low ELISA sensitivity (50%), but the sensitivity was significantly increased (up to 92.8%) in serum samples with antibody titres up to 5 EU/mL. Studies have shown that the sensitivity of the Bio-Rad ELISA for fox serum samples from ORV regions is significantly lower (32.4%) than RFFIT [35]. In addition, similar tests in EU-RL in Nancy[36] showed that ELISA antibody titres (EU/mL) in control samples were two to five times lower than classical virus-neutralisation assays (SNT-serum neutralization test). Bio-Rad ELISA results only correlate reasonably well with RFFIT when the expected antibody titres are greater than 1.0 IU/mL. However, when the expected antibody titre is lower, its sensitivity is completely inadequate. Therefore, the use of Bio-Rad ELISA should rely on its specificity to identify seronegative specimens, since its sensitivity in determining antibody titres in vaccinated wildlife animals should be deemed unsatisfactory [39]**.** Therefore, the lack of sensitivity of the Bio-Rad PlateliaTM Rabies II kit and a certain degree of variability in the quality of field sera samples could be a key point for identifying threshold seroconversion in target wildlife during an ORV programmes. Consequently, the evaluation of the Lithuanian ORV programme in low immunogenic serum samples and predictably weak (lower than 50%) ELISA kit sensitivity can influence the interpretation of the seroprevalence rate in target populations.

## Conclusions

The long-term ORV programme has been an effective tool in both RF and RD populations in Lithuania – only three rabies cases identified in buffer zones since 2015 (Fig. 3). 14–29% of RF and 7–25% of RD serum samples were identified as ELISA-negative, but with low 0.125 < 0.49 EU/mL Abs titres. Comparative serological data indicate a sensitivity less than 50% of ELISA tests in poor quality serum samples with low antibody titers, therefore it can be expected that the part of Lithuanian samples collected from vaccination areas in the field and tested by ELISA could be considered negative due to their poor biological quality. The real seroconversion percentage in susceptible animal populations could be higher: RF and RD with low ELISA Abs titres (specifically 10% serum samples within 0.4 < 0.49 EU/mL) likely participated in the formation of the overall immune status of the population. Including target animals with low Abs could directly influence the interpretation of oral vaccination efficacy.

## Supplementary Information

**Additional file 1.** Seroconversion (ELISA Abs titres EU/mL; %) in adult (A) and juvenile (J) red fox (RF) subpopulations during the 2010–2019 ORV programme in Lithuania.

**Additional file 2.** Seroconversion (ELISA Abs titres EU/mL; %) in adult (A) / juvenile (J) raccoon dog (RD) subpopulations during the 2010–2019 ORV programme in Lithuania.

**Additional file 3.** Comparative analysis of 2010–2019 ORV seroconversion (pos.% at ≥ 0.5 EU/mL) in different age groups of red foxes (RF) and raccoon dogs (RD).

**Additional file 4.** Seroconversion (ELISA Abs titres EU/mL; %) in Lithuanian red foxes (RF) during the 2010–2019 ORV spring (S) and autumn (A) vaccination periods.

**Additional file 5.** Seroconversion (ELISA Abs titres EU/mL; %) in Lithuanian raccoon dogs (RD) during the 2010–2019 ORV spring (S) and autumn (A) vaccination period.

**Additional file 6.** Comparative analysis of seroconversion (pos.% at ≥ 0.5 EU/mL) of red foxes (RF) and raccoon dogs (RD) in the 2010–2019 ORV spring and autumn campaigns.

## Data Availability

The datasets used and/or analyzed during the current study are available from the corresponding author on reasonable request.

## Abbreviations

95% CI: 95% Confidence interval
EFSA: European Food Safety Authority
ELISA: Enzyme-linked immunosorbent assay
EU/mL: Equivalent units per millilitre
FAVN: Fluorescent antibody virus neutralization test
FFU: Focus forming units
FUCHSORAL (IDT Biologika GmbH, Germany): Live attenuated RV vaccine
LYSVULPEN (Bioveta A.S., Czech Republic): Live attenuated RV vaccine
M: Mean values
NFVRAI: National Food and Veterinary Risk Assessment Institute ofLithuania
OD: Optical density
ORV: Oral rabies vaccination
*p*: Confidence factor
PC: Calibrated positive controls
*r*: Spearman correlation coefficient
RD: Raccoon dog (*Nyctereutes procyonoides)*
RF: Red fox (*Vulpes vulpes*)
RFFIT: Rapid fluorescent focus inhibition test
RV: Rabies virus
S1–S6: Quantitative standards
SD: Average standard deviation
SNT: Serum neutralization test
TCID: Tissue culture infective dose
WHO: World Health Organization
