# Supplementary material for: Analysis of seroprevalence in target wildlife during the oral rabies vaccination programme in Lithuania
Source: Acta Vet Scand. 2021 Mar 20;63:12. doi: 10.1186/s13028-021-00577-z (PMC7981835; doi:10.1186/s13028-021-00577-z)
Supplement: Supplementary file 1 — Additional file 1. Seroconversion (ELISA Abs titres EU/mL; %) in adult (A) and juvenile (J) red fox (RF) subpopulations during the 2010–2019 ORV programme in Lithuania. [file 13028_2021_577_MOESM1_ESM.doc]

**Additional file 1.** Seroconversion (ELISA Abs titres EU/mL; %) in adult (A) and juvenile (J) red fox (RF) subpopulation during the 2010-2019 ORV programme in Lithuania

| **ORV Period** | **2010**  **J** | **2010**  **A** | **2011**  **J** | **2011**  **A** | **2012**  **J** | **2012**  **A** | **2013**  **J** | **2013**  **A** | **2014**  **J** | **2014**  **A** | **2015**  **J** | **2015**  **A** | **2016**  **J** | **2016**  **A** | **2017**  **J** | **2017**  **A** | **2018**  **J** | **2018**  **A** | **2019**  **J** | **2019**  **A** |
| --- | --- | --- | --- | --- | --- | --- | --- | --- | --- | --- | --- | --- | --- | --- | --- | --- | --- | --- | --- | --- |
| **Samples (n)** | **92** | **547** | **110** | **670** | **138** | **720** | **130** | **681** | **166** | **834** | **95** | **478** | **48** | **856** | **43** | **847** | **66** | **383** | **39** | **317** |
| **<0.125 EU/mL** | **55.5** | **48.7** | **37.7** | **33.2** | **54.5** | **51.3** | **63.8** | **48.5** | **58.9** | **53.6** | **33.1** | **24.5** | **42.2** | **35.9** | **41.7** | **35.7** | **45.8** | **39.8** | **53.1** | **48.8** |
| **<95 CI** | 37.0 | 32.9 | 22.3 | 17.7 | 39.8 | 35.7 | 49.9 | 32.5 | 44.0 | 39.5 | 17.5 | 10.0 | 26.6 | 20.4 | 26.4 | 20.1 | 29.5 | 24.7 | 37.2 | 33.3 |
| **95CI <** | 74.1 | 64.5 | 53.2 | 48.9 | 69.2 | 67.2 | 77.5 | 64.4 | 73.8 | 68.5 | 48.6 | 39.0 | 57.8 | 51.4 | 57.3 | 51.3 | 62.2 | 54.9 | 69.1 | 64.7 |
| **0.125<0.49 EU/mL** | **22.5** | **20.6** | **26.5** | **21.4** | **26.5** | **27.3** | **24.2** | **24.4** | **30.2** | **31.5** | **19.3** | **24.9** | **19.6** | **23.7** | **24.3** | **23.5** | **16** | **19.5** | **13.6** | **17.6** |
| **<95 CI** | 8.5 | 6.3 | 12.7 | 7.4 | 12.6 | 13.4 | 10.2 | 10.6 | 15.1 | 16.2 | 4.8 | 10.9 | 6.2 | 9.6 | 10.4 | 9.5 | 2.0 | 6.1 | 2.7 | 4.2 |
| **95CI <** | 36.5 | 34.8 | 40.3 | 35.6 | 40.4 | 41.2 | 38.1 | 38.3 | 45.4 | 46.6 | 33.7 | 38.9 | 33.2 | 37.5 | 38.1 | 37.4 | 30.2 | 32.9 | 24.7 | 30.8 |
| **0.5≤2 EU/mL** | **18** | **25.1** | **29.6** | **28.5** | **19** | **15.9** | **10.1** | **22.8** | **10.1** | **10.4** | **37.9** | **34.1** | **30.4** | **27.7** | **25.5** | **30.2** | **31.5** | **27.8** | **28.5** | **26.7** |
| **<95 CI** | 4.6 | 11.3 | 14.9 | 14.4 | 5.7 | 3.0 | 0.5 | 8.8 | 0.6 | 0.9 | 21.5 | 17.8 | 15.1 | 13.8 | 11.5 | 14.5 | 16.0 | 13.9 | 14.4 | 12.0 |
| **95CI <** | 31.3 | 40.2 | 44.3 | 42.7 | 32.3 | 29.1 | 19.6 | 36.8 | 19.7 | 19.8 | 54.2 | 50.6 | 47.8 | 41.8 | 39.5 | 46.0 | 47.1 | 41.9 | 42.7 | 41.4 |
| **>2 EU/mL** | **4** | **5.5** | **6.1** | **16.9** | **0** | **5.4** | **2.01** | **4.3** | **0.72** | **4.6** | **9.6** | **16.5** | **7.8** | **12.7** | **8.5** | **10.6** | **6.7** | **12.9** | **4.8** | **6.9** |
| **<95 CI** | 0.6 | 0.9 | 0.6 | 2.7 | - | 1.1 | 1.2 | 1.8 | 0.3 | 1.7 | 0.3 | 3.5 | 0.2 | 2.4 | 0.5 | 1.2 | 0.2 | 2.3 | 1.5 | 0.9 |
| **95CI <** | 8.8 | 12.0 | 12.8 | 31.2 | - | 11.8 | 5.2 | 10.6 | 1.9 | 10.7 | 19.1 | 29.6 | 15.6 | 23.1 | 16.6 | 20.0 | 13.5 | 23.5 | 11.0 | 12.9 |
