# Supplementary material for: Analysis of seroprevalence in target wildlife during the oral rabies vaccination programme in Lithuania
Source: Acta Vet Scand. 2021 Mar 20;63:12. doi: 10.1186/s13028-021-00577-z (PMC7981835; doi:10.1186/s13028-021-00577-z)
Supplement: Supplementary file 2 — Additional file 2. Seroconversion (ELISA Abs titres EU/mL; %) in adult (A) / juvenile (J) raccoon dog (RD) subpopulations during the 2010–2019 ORV programme in Lithuania. [file 13028_2021_577_MOESM2_ESM.doc]

**Additional file 2.** Seroconversion (ELISA Abs titres EU/mL; %) in adult (A) / juvenile (J) raccoon dog (RD) subpopulations during the 2010-2019 ORV programme in Lithuania

| **ORV Period** | **2010**  **J** | **2010**  **A** | **2011**  **J** | **2011**  **A** | **2012**  **J** | **2012**  **A** | **2013**  **J** | **2013**  **A** | **2014**  **J** | **2014**  **A** | **2015**  **J** | **2015**  **A** | **2016**  **J** | **2016**  **A** | **2017**  **J** | **2017**  **A** | **2018**  **J** | **2018**  **A** | **2019**  **J** | **2019**  **A** |
| --- | --- | --- | --- | --- | --- | --- | --- | --- | --- | --- | --- | --- | --- | --- | --- | --- | --- | --- | --- | --- |
| **Samples (n)** | **75** | **433** | **46** | **164** | **45** | **171** | **40** | **143** | **70** | **256** | **46** | **148** | **18** | **179** | **10** | **159** | **10** | **66** | **14** | **53** |
| **<0.125 EU/ml** | **77.8** | **56.5** | **66.2** | **45.9** | **64.3** | **57.5** | **58.2** | **37.4** | **68.7** | **44.1** | **24.6** | **24.5** | **51.1** | **41.7** | **59.6** | **39.9** | **29.9** | **48.1** | **38.1** | **40.1** |
| **<95 CI** | 64.1 | 45.3 | 50.1 | 33.3 | 49.4 | 46.8 | 47.9 | 22.1 | 55.9 | 30.0 | 14.3 | 16.5 | 39.1 | 28.3 | 60.0 | 23.5 | 18.7 | 35.5 | 24.0 | 29.9 |
| **95CI <** | 86.7 | 68.2 | 78.9 | 66.5 | 77.7 | 70.7 | 72.0 | 50.3 | 83.3 | 55.1 | 37.2 | 32.9 | 69.7 | 57.6 | 70.9 | 49.3 | 40.9 | 60.0 | 48.9 | 55.5 |
| **0.125<0.49 EU/ml** | **11.1** | **6.5** | **15.6** | **17.9** | **14.2** | **20.6** | **18.6** | **25.2** | **6.3** | **24.1** | **24.6** | **10.9** | **20** | **16.5** | **13.7** | **18.2** | **20.1** | **15.7** | **11.9** | **24.2** |
| **<95 CI** | 2.5 | -0.7 | 7.2 | 9.0 | 4.2 | 10.3 | 9.1 | 13.9 | -0.6 | 11.8 | 14.2 | 2.3 | 11.2 | 7.9 | 4.0 | 8.5 | 11.5 | 7.1 | 2.9 | 9.9 |
| **95CI <** | 19.4 | 13.4 | 24.1 | 26.1 | 24.4 | 32.0 | 28.3 | 35.9 | 13.9 | 33.3 | 35.0 | 17.0 | 29.6 | 25.1 | 22.2 | 28.1 | 30.0 | 24.3 | 21.1 | 34.3 |
| **0.5≤2 EU/ml** | **11.1** | **28.3** | **12.9** | **20.4** | **19.1** | **15.7** | **13.9** | **27.6** | **15.6** | **20** | **43.4** | **33.5** | **15.6** | **28.1** | **17** | **22.7** | **34.2** | **24** | **33.1** | **23.7** |
| **<95 CI** | 2.3 | 15.5 | 5.6 | 10.3 | 9.7 | 6.8 | 5.3 | 15.3 | 6.9 | 10.9 | 31.1 | 22.3 | 6.9 | 16.0 | 8.8 | 9.5 | 20.1 | 10.9 | 19.9 | 11.9 |
| **95CI <** | 18.9 | 37.7 | 20.3 | 30.5 | 28.6 | 24.2 | 22.4 | 39.9 | 24.2 | 29.3 | 58.3 | 53.1 | 24.3 | 40.3 | 25.3 | 33.5 | 49.4 | 35.2 | 45.8 | 35.6 |
| **>2 EU/ml** | **0** | **8.7** | **5.2** | **15.8** | **2.4** | **6.2** | **9.3** | **9.8** | **9.4** | **11.8** | **7.2** | **31.1** | **13.3** | **13.7** | **9.7** | **19.2** | **15.8** | **12.2** | **16.9** | **12** |
| **<95 CI** | - | 0.4 | 1.9 | 7.3 | 0.6 | 0.6 | 1.8 | 2.1 | 2.0 | 3.0 | 0.8 | 19.5 | 3.2 | 5.5 | 2.2 | 9.9 | 7.3 | 3.8 | 7.5 | 3.6 |
| **95CI <** | - | 16.4 | 12.8 | 24.5 | 5.1 | 12.9 | 17.9 | 19.7 | 16.9 | 20.1 | 14.5 | 42.9 | 22.7 | 21.8 | 15.0 | 28.5 | 24.2 | 20.5 | 26.3 | 20.1 |
