# Supplementary material for: Analysis of seroprevalence in target wildlife during the oral rabies vaccination programme in Lithuania
Source: Acta Vet Scand. 2021 Mar 20;63:12. doi: 10.1186/s13028-021-00577-z (PMC7981835; doi:10.1186/s13028-021-00577-z)
Supplement: Supplementary file 3 — Additional file 3. Comparative analysis of 2010–2019 ORV seroconversion (pos.% at ≥ 0.5 EU/mL) in different age groups of red foxes (RF) and raccoon dogs (RD). [file 13028_2021_577_MOESM3_ESM.doc]

**Additional file 3.** Comparative analysis of 2010-2019 ORV seroconversion (pos.% at ≥0.5 EU/mL) in different age groups of

red foxes (RF) and raccoon dogs (RD)

| **ORV Period** | **2010** | **2011** | **2012** | **2013** | **2014** | **2015** | **2016** | **2017** | **2018** | **2019** |
| --- | --- | --- | --- | --- | --- | --- | --- | --- | --- | --- |
| **Samples RF (n)** | **639** | **780** | **858** | **811** | **1000** | **574** | **904** | **890** | **449** | **356** |
| **RF juvenile** | **22.2** | **35.7** | **19** | **12.1** | **10.8** | **47.6** | **38.2** | **34.1** | **38.2** | **33.3** |
| **<95 CI** | 9.1 | 20.5 | 2.8 | 3.0 | 2.4 | 28.6 | 22.7 | 18.9 | 22.8 | 18.6 |
| **95CI <** | 34.5 | 51.0 | 35.3 | 21.5 | 19.2 | 66.7 | 53.7 | 49.3 | 53.7 | 48.0 |
| **RF adult** | **30.6** | **45.6** | **25** | **27.1** | **15** | **50.6** | **40.4** | **40.1** | **40.7** | **33.6** |
| **<95 CI** | 12.8 | 26.6 | 11.1 | 13.6 | 1.6 | 38.6 | 26.1 | 27.7 | 26.6 | 19.1 |
| **95CI <** | 48.3 | 64.6 | 38.9 | 40.5 | 28.5 | 62.6 | 54.6 | 52.5 | 54.7 | 48.1 |
| **Samples RD (n)** | **508** | **210** | **216** | **183** | **326** | **194** | **197** | **169** | **76** | **67** |
| **RD juvenile** | **11.2** | **18.2** | **21.5** | **23.3** | **25** | **50.7** | **28.9** | **26.7** | **50** | **50** |
| **<95 CI** | 2.6 | 1.9 | 8.2 | 9.1 | 11.2 | 32.9 | 15.4 | 13.3 | 31.7 | 31.8 |
| **95CI <** | 19.8 | 34.9 | 34.7 | 37.6 | 38.9 | 68.7 | 42.4 | 40.1 | 68.2 | 68.3 |
| **RD adult** | **36.9** | **36.2** | **22.0** | **37.4** | **31.7** | **64.6** | **41.7** | **41.9** | **36.2** | **35.7** |
| **<95 CI** | 20.7 | 19.8 | 8.9 | 21.6 | 17.1 | 47.1 | 27.0 | 27.4 | 21.5 | 20.4 |
| **95CI <** | 53.1 | 50.4 | 35.2 | 53.2 | 46.3 | 82.1 | 56.5 | 56.4 | 51.2 | 50.9 |
