# Supplementary material for: Analysis of seroprevalence in target wildlife during the oral rabies vaccination programme in Lithuania
Source: Acta Vet Scand. 2021 Mar 20;63:12. doi: 10.1186/s13028-021-00577-z (PMC7981835; doi:10.1186/s13028-021-00577-z)
Supplement: Supplementary file 4 — Additional file 4. Seroconversion (ELISA Abs titres EU/mL; %) in Lithuanian red foxes (RF) during the 2010–2019 ORV spring (S) and autumn (A) vaccination periods. [file 13028_2021_577_MOESM4_ESM.doc]

**Additional file 4.** Seroconversion (ELISA Abs titres EU/mL; %) in Lithuanian red foxes (RF) during the 2010-2019 ORV spring (S) and autumn (A) vaccination periods

| **ORV Period** | **2010**  **S** | **2010**  **A** | **2011**  **S** | **2011**  **A** | **2012**  **S** | **2012**  **A** | **2013**  **S** | **2013**  **A** | **2014**  **S** | **2014**  **A** | **2015**  **S** | **2015**  **A** | **2016**  **S** | **2016**  **A** | **2017**  **S** | **2017**  **A** | **2018**  **S** | **2018**  **A** | **2019**  **S** | **2019**  **A** |
| --- | --- | --- | --- | --- | --- | --- | --- | --- | --- | --- | --- | --- | --- | --- | --- | --- | --- | --- | --- | --- |
| **Samples (n)** | **49** | **590** | **500** | **280** | **418** | **440** | **687** | **124** | **516** | **484** | **455** | **119** | **427** | **477** | **562** | **328** | **265** | **184** | **219** | **137** |
| **<0.125 EU/ml** | **50** | **51.1** | **24** | **34.6** | **45.6** | **60.8** | **46.5** | **55.3** | **55.1** | **50.4** | **17.5** | **35** | **29.9** | **60.9** | **43.5** | **23.5** | **51.8** | **39** | **49.2** | **52** |
| **<95 CI** | 34.6 | 35.1 | 8.9 | 17.0 | 26.1 | 46.1 | 26.5 | 35.0 | 31.3 | 30.7 | 1.6 | 18.2 | 12.5 | 42.4 | 23.9 | 6.5 | 32.3 | 21.0 | 30.1 | 32.1 |
| **95CI <** | 66.1 | 67.0 | 40.1 | 52.1 | 65.0 | 75.6 | 64.2 | 75.1 | 70.4 | 70.1 | 33.5 | 52.1 | 46.4 | 79.4 | 63.5 | 40.3 | 71.2 | 56.9 | 68.4 | 71.7 |
| **0.125<0.49 EU/ml** | **21.9** | **20.2** | **18** | **22.5** | **29.6** | **21.9** | **25.6** | **23.2** | **32.1** | **26.4** | **27.2** | **20.5** | **23.4** | **21.7** | **28** | **23.9** | **17.5** | **16.4** | **13.6** | **18.1** |
| **<95 CI** | 4.5 | 10.2 | 8.9 | 10.5 | 17.1 | 11.0 | 14.3 | 6.0 | 19.2 | 14.4 | 16.5 | 10.3 | 12.5 | 11.0 | 17.5 | 12.5 | 1.9 | 5.7 | 4.9 | 7.5 |
| **95CI <** | 39.3 | 30.1 | 27.1 | 34.1 | 41.0 | 32.7 | 36.9 | 40.1 | 45.1 | 38.4 | 38.1 | 30.8 | 34.2 | 32.4 | 38.5 | 35.5 | 33.5 | 27.1 | 22.4 | 28.5 |
| **0.5≤2 EU/ml** | **23.4** | **22.3** | **44** | **27.4** | **18.3** | **15.9** | **26.1** | **15.6** | **8.7** | **20** | **38.2** | **31.1** | **33.1** | **11.6** | **18.5** | **40.2** | **22.5** | **32.5** | **28.7** | **23.1** |
| **<95 CI** | 11.8 | 10.5 | 27.2 | 16.8 | 7.7 | 6.1 | 11.5 | 5.7 | 2.7 | 8.8 | 21.4 | 15.2 | 17.8 | 3.5 | 7.6 | 23.1 | 10.7 | 17.6 | 16.5 | 11.9 |
| **95CI <** | 35.1 | 34.2 | 60.9 | 38.1 | 28.9 | 25.8 | 40.4 | 25.3 | 14.4 | 31.1 | 54.9 | 46.6 | 48.2 | 19.7 | 29.4 | 57.5 | 34.4 | 47.5 | 41.1 | 34.2 |
| **>2 EU/ml** | **4.7** | **6.4** | **14** | **15.5** | **6.5** | **1.4** | **1.8** | **5.9** | **4.1** | **3.2** | **17.1** | **13.4** | **13.6** | **5.8** | **10** | **12.4** | **8.2** | **12.1** | **8.5** | **6.8** |
| **<95 CI** | 0.4 | 1.2 | 3.6 | 4.4 | 1.3 | 1.1 | 1.2 | 1.3 | 0.4 | 0.6 | 7.4 | 5.2 | 5.3 | 1.1 | 2.4 | 4.2 | 2.5 | 3.8 | 2.7 | 1.4 |
| **95CI <** | 9.8 | 11.6 | 24.4 | 26.7 | 11.7 | 4.0 | 4.8 | 10.4 | 7.9 | 7.0 | 22.5 | 21.6 | 21.9 | 10.3 | 17.8 | 20.6 | 13.9 | 20.4 | 14.2 | 12.4 |
