# Supplementary material for: Analysis of seroprevalence in target wildlife during the oral rabies vaccination programme in Lithuania
Source: Acta Vet Scand. 2021 Mar 20;63:12. doi: 10.1186/s13028-021-00577-z (PMC7981835; doi:10.1186/s13028-021-00577-z)
Supplement: Supplementary file 5 — Additional file 5. Seroconversion (ELISA Abs titres EU/mL; %) in Lithuanian raccoon dogs (RD) during the 2010–2019 ORV spring (S) and autumn (A) vaccination period. [file 13028_2021_577_MOESM5_ESM.doc]

**Additional file 5.** Seroconversion (ELISA Abs titres EU/mL; %) in Lithuanian raccoon dogs (RD) during the 2010-2019 ORV spring (S) and autumn (A) vaccination period

| **ORV Period** | **2010**  **S** | **2010**  **A** | **2011**  **S** | **2011**  **A** | **2012**  **S** | **2012**  **A** | **2013**  **S** | **2013**  **A** | **2014**  **S** | **2014**  **A** | **2015**  **S** | **2015**  **A** | **2016**  **S** | **2016**  **A** | **2017**  **S** | **2017**  **A** | **2018**  **S** | **2018**  **A** | **2019**  **S** | **2019**  **A** |
| --- | --- | --- | --- | --- | --- | --- | --- | --- | --- | --- | --- | --- | --- | --- | --- | --- | --- | --- | --- | --- |
| **Samples (n)** | **60** | **448** | **117** | **93** | **87** | **129** | **98** | **85** | **169** | **157** | **131** | **63** | **129** | **68** | **104** | **65** | **45** | **31** | **40** | **25** |
| **<0.125 EU/ml** | **62.1** | **57.7** | **39.7** | **50.2** | **55.1** | **64.2** | **32.5** | **48.8** | **59.1** | **26.9** | **20.7** | **23.8** | **38.8** | **55.2** | **44.8** | **29.9** | **49.9** | **34.6** | **40.9** | **55.2** |
| **<95 CI** | 32.8 | 39.1 | 29.5 | 33.3 | 40.3 | 54.5 | 2404 | 36.1 | 42.9 | 13.9 | 9.5 | 10.7 | 25.0 | 40.0 | 30.5 | 20.0 | 39.3 | 20.1 | 29.6 | 40.0 |
| **95CI <** | 80.4 | 75.2 | 58.8 | 68.3 | 69.9 | 84.7 | 49.0 | 59.1 | 76.5 | 35.3 | 33.1 | 33.9 | 49.1 | 66.6 | 58.1 | 38.9 | 60.6 | 55.3 | 59.3 | 70.1 |
| **0.125<0.49 EU/ml** | **6.9** | **7.7** | **12.1** | **18.2** | **20.6** | **17.3** | **23.5** | **24.4** | **21.8** | **20.7** | **14.8** | **13.6** | **17.5** | **17.2** | **29.5** | **14.5** | **19.4** | **23.2** | **14.9** | **15** |
| **<95 CI** | 1.1 | 0.9 | 2.3 | 9.9 | 12.9 | 10.1 | 11.0 | 11.8 | 13.3 | 10.0 | 7.9 | 5.9 | 8.8 | 9.3 | 15.9 | 7.7 | 11.2 | 11.8 | 7.9 | 5.9 |
| **95CI <** | 15.9 | 18.2 | 27.7 | 33.5 | 33.3 | 25.8 | 34.4 | 38.5 | 29.9 | 30.1 | 23.9 | 21.5 | 27.7 | 30.0 | 39.1 | 28.8 | 30.0 | 35.1 | 29.0 | 28.8 |
| **0.5<2 EU/ml** | **24.1** | **26.9** | **24.1** | **18.7** | **15.9** | **17.3** | **32.5** | **17.9** | **13.6** | **30.2** | **36.7** | **36.7** | **27.8** | **18.9** | **15.6** | **42.8** | **21.8** | **30.8** | **28.4** | **16** |
| **<95 CI** | 18.5 | 19.8 | 10.1 | 8.2 | 8.5 | 8.9 | 19.9 | 10.1 | 8.2 | 19.7 | 25.9 | 30.3 | 19.8 | 10.2 | 7.9 | 29.9 | 10.9 | 20.3 | 20.0 | 8.9 |
| **95CI <** | 33.3 | 35.0 | 39.8 | 31.2 | 29.9 | 27.8 | 40.3 | 25.5 | 21.4 | 41.8 | 50.0 | 46.3 | 39.1 | 28.9 | 27.1 | 59.3 | 32.9 | 41.1 | 38.9 | 23.4 |
| **>2 EU/ml** | **6.9** | **7.7** | **24.1** | **12.9** | **8.4** | **1.2** | **11.5** | **8.9** | **5.5** | **22.2** | **27.8** | **25.9** | **15.9** | **8.7** | **10.1** | **12.8** | **8.9** | **11.4** | **15.8** | **13.8** |
| **<95 CI** | 0.5 | 1.4 | 8.9 | 3.8 | 2.3 | 0.9 | 1.1 | 0.3 | 2.2 | 11.9 | 20.0 | 18.2 | 10.1 | 1.2 | 2.1 | 2.0 | 0.7 | 0.1 | 3.9 | 4.0 |
| **95CI <** | 15.5 | 8.3 | 33.3 | 25.5 | 12.5 | 5.9 | 22.9 | 17.6 | 8.8 | 30.3 | 37.8 | 33.3 | 28.5 | 12.7 | 20.0 | 25.0 | 18.8 | 22.4 | 24.7 | 20.3 |
|  |  |  |  |  |  |  |  |  |  |  |  |  |  |  |  |  |  |  |  |  |
