# Supplementary material for: Analysis of seroprevalence in target wildlife during the oral rabies vaccination programme in Lithuania
Source: Acta Vet Scand. 2021 Mar 20;63:12. doi: 10.1186/s13028-021-00577-z (PMC7981835; doi:10.1186/s13028-021-00577-z)
Supplement: Supplementary file 6 — Additional file 6. Comparative analysis of seroconversion (pos.% at ≥ 0.5 EU/mL) of red foxes (RF) and raccoon dogs (RD) in the 2010–2019 ORV spring and autumn campaigns. [file 13028_2021_577_MOESM6_ESM.doc]

**Additional file 6.** Comparative analysis of seroconversion (pos.% at ≥0.5 EU/mL) of red foxes (RF) and raccoon dogs (RD) in the 2010-2019 ORV spring and autumn campaigns

| **ORV Period** | **2010** | **2011** | **2012** | **2013** | **2014** | **2015** | **2016** | **2017** | **2018** | **2019** |
| --- | --- | --- | --- | --- | --- | --- | --- | --- | --- | --- |
| **Samples RF (n)** | **639** | **780** | **858** | **811** | **1000** | **574** | **904** | **890** | **449** | **356** |
| **RF spring** | **28.1** | **58** | **24.8** | **27.9** | **12.8** | **55.3** | **46.8** | **28.5** | **30.7** | **37.2** |
| **<95 CI** | 9.7 | 40.8 | 7.3 | 9.7 | 1.5 | 36.5 | 26.3 | 9.2 | 10.2 | 16.6 |
| **95CI <** | 46.5 | 75.4 | 42.2 | 46.2 | 24.0 | 74.0 | 67.4 | 47.9 | 51.2 | 57.8 |
| **RF autumn** | **28.7** | **42.9** | **42.9** | **21.5** | **23.2** | **44.6** | **17.4** | **52.6** | **44.6** | **29.9** |
| **<95 CI** | 9.2 | 21.2 | 21.1 | 4.4 | 5.6 | 25.2 | 4.3 | 33.3 | 25.3 | 9.3 |
| **95CI <** | 48.2 | 64.8 | 64.7 | 38.6 | 40.7 | 63.9 | 30.5 | 71.9 | 64.0 | 50.5 |
| **Samples RD (n)** | **508** | **210** | **216** | **183** | **326** | **194** | **197** | **169** | **76** | **67** |
| **RD spring** | **31.1** | **48.3** | **24.3** | **43.8** | **19.1** | **64.5** | **43.6** | **25.7** | **30.7** | **44.2** |
| **<95 CI** | 10.8 | 25.6 | 7.1 | 22.3 | 5.4 | 48.5 | 22.0 | 8.3 | 10.3 | 23.1 |
| **95CI <** | 51.5 | 71.0 | 41.6 | 65.3 | 32.8 | 80.6 | 65.1 | 43.1 | 51.3 | 65.4 |
| **RD autumn** | **34.6** | **31.6** | **18.5** | **26.8** | **52.4** | **62.6** | **27.6** | **55.6** | **42.2** | **29.8** |
| **<95 CI** | 14.4 | 12.8 | 5.1 | 9.1 | 33.1 | 46.3 | 9.5 | 37.1 | 22.1 | 9.2 |
| **95CI <** | 56.8 | 50.4 | 31.8 | 44.4 | 71.7 | 78.9 | 45.6 | 74.1 | 62.3 | 50.4 |
